# Medical Obituaries

**Published:** 1854-03

**Authors:** 


From the Iowa Medical Journal.

*Medical Obituaries.*

Died at Philadelphia, January 4th, Dr. Samuel McClellan, aged 54 years. He was brother of Dr. George McClellan, the late distinguished surgeon of that city. Both brothers lectured in the Jefferson College from its commencement, in which Dr. Samuel McClellan was formerly Professor of Obsterics, though he afterwards lectured on the same branch in the Pennsylvania College. He was in the enjoyment of an extensive obstetric practice, and his death is said to have been owing to fatigue from over-exertion. His death was much regretted by his numerous friends, both public and professional.

Died, Dr. John Esten Cooke, on the 30th of October, in the 70th year of his age. Dr. Cook succeeded Dr. Drake in the Chair of the Theory and Practice in Transylvania University, and was subsequently connected with the Medical Institute of Louisville. He was a voluminous writer. Besides his treatise on Pathology and Therapeutics, he wrote for the American Medical Recorder, the Transylvania Journal of Medicine, and occasionally on theological subjects.

Drs. Drake and Cooke were fellow students in Philadelphia in 1805.

It is with profound sorrow and deep-felt regret, that we are compelled to record the untimely death of R. L. Howard, M. D., Professor of Surgery in the Starling Medical College of Columbus, Ohio. He died recently, of Pneumonia complicated (says a private letter,) with Bright's disease of the Kidney. We have known him long and well, and can testify to his sterling qualities as a man, his rare acquirements and success as a physician and surgeon, and his abilities as an instructor. The place made vacant by his death, it will be very difficult to fill, either in the flourishing institution with which he was connected, in the ranks of the profession, or in the community in which he lived and labored.

From the New Hampshire Journal of Medicine.

Died, Dec. 1, 1853, at New Orleans, of Epidemic Cholera, Abner Hester, M. D., the talented editor of the New Orleans Medical and Surgical Journal, aged 40 years. Dr. Hester enjoyed a high reputation as a skilful and experienced practitioner, and a zealous cultivator of our science. His premature death will be a serious loss to the profession.

Died, in New York, on the 7th of December last, aged 62 years, Thomas G. Mower, M. D., one of the senior surgeons of the United States Army, and a gentleman of varied scientific attainments.

Died, in Paris, an the 2nd of October, of diabetes, complicated with albuminuria, M. Argo, Perpetual Secretary of the Academy of Sciences, and Associate Member of the Academy of Medicine, aged 67 years and a half.

Died, in London, on the 27th of December last, James Gilchrist, M. D., Inspector General of the Army hospitals, and Corresponding Member of the National Academy of Medicine of France.

